# Impaired Mitochondrial Respiration in Upper Compared to Lower Body Differentiated Human Adipocytes and Adipose Tissue

**DOI:** 10.1210/clinem/dgae086

**Published:** 2024-02-20

**Authors:** Ioannis G Lempesis, Nicole Hoebers, Yvonne Essers, Johan W E Jocken, Ludwig J Dubois, Ellen E Blaak, Konstantinos N Manolopoulos, Gijs H Goossens

**Affiliations:** Institute of Metabolism and Systems Research (IMSR), College of Medical and Dental Sciences, University of Birmingham, Birmingham B15 2TT, UK; Centre for Endocrinology, Diabetes and Metabolism, Birmingham Health Partners, Birmingham B15 2TT, UK; Department of Human Biology, NUTRIM School of Nutrition and Translational Research in Metabolism, Maastricht University Medical Centre+, 6200 MD Maastricht, The Netherlands; Department of Human Biology, NUTRIM School of Nutrition and Translational Research in Metabolism, Maastricht University Medical Centre+, 6200 MD Maastricht, The Netherlands; Department of Human Biology, NUTRIM School of Nutrition and Translational Research in Metabolism, Maastricht University Medical Centre+, 6200 MD Maastricht, The Netherlands; Department of Human Biology, NUTRIM School of Nutrition and Translational Research in Metabolism, Maastricht University Medical Centre+, 6200 MD Maastricht, The Netherlands; The M-Lab, Department of Precision Medicine, GROW School for Oncology and Reproduction, Maastricht University Medical Centre+, 6200 MD Maastricht, The Netherlands; Department of Human Biology, NUTRIM School of Nutrition and Translational Research in Metabolism, Maastricht University Medical Centre+, 6200 MD Maastricht, The Netherlands; Institute of Metabolism and Systems Research (IMSR), College of Medical and Dental Sciences, University of Birmingham, Birmingham B15 2TT, UK; Centre for Endocrinology, Diabetes and Metabolism, Birmingham Health Partners, Birmingham B15 2TT, UK; Department of Human Biology, NUTRIM School of Nutrition and Translational Research in Metabolism, Maastricht University Medical Centre+, 6200 MD Maastricht, The Netherlands

**Keywords:** adipose tissue, body fat distribution, mitochondria, obesity, oxygen consumption

## Abstract

**Context:**

Abdominal obesity is associated with increased cardiometabolic disease risk, while lower body fat seems to confer protection against obesity-related complications. The functional differences between upper and lower body adipose tissue (AT) remain poorly understood.

**Objective:**

We aimed to examine whether mitochondrial respiration is impaired in abdominal as compared to femoral differentiated human multipotent adipose-derived stem cells (hMADS; primary outcome) and AT in postmenopausal women.

**Design:**

In this cross-sectional study, 23 postmenopausal women with normal weight or obesity were recruited at the University of Birmingham/Queen Elizabeth Hospital Birmingham (Birmingham, UK). We collected abdominal and femoral subcutaneous AT biopsies to determine mitochondrial oxygen consumption rates in differentiated abdominal and femoral hMADS. Furthermore, we assessed oxidative phosphorylation (OXPHOS) protein expression and mitochondrial DNA (mtDNA) content in abdominal and femoral AT as well as hMADS. Finally, we explored in vivo fractional oxygen extraction and carbon dioxide release across abdominal and femoral subcutaneous AT in a subgroup of the same individuals with normal weight or obesity.

**Results:**

We found lower basal and maximal uncoupled mitochondrial oxygen consumption rates in abdominal compared to femoral hMADS. In line, in vivo fractional oxygen extraction and carbon dioxide release were lower across abdominal than femoral AT. OXPHOS protein expression and mtDNA content did not significantly differ between abdominal and femoral differentiated hMADS and AT.

**Conclusion:**

The present findings demonstrate that in vitro mitochondrial respiration and in vivo oxygen fractional extraction are less in upper compared to lower body differentiated hMADS and AT, respectively, in postmenopausal women.

Body fat distribution is closely associated with the risk for developing cardiometabolic diseases, with lower body (gluteofemoral) adipose tissue (AT) possessing protective properties (1-6). This may imply that depot differences in AT function contribute to the cardiometabolic disease risk associated with a certain body fat distribution pattern. It is well established that AT dysfunction in obesity is characterized by adipocyte hypertrophy, impairments in lipid metabolism, cellular stress, inflammation, and altered oxygenation due to blunted AT blood flow (ATBF) and/or mitochondrial impairments, together promoting the development of cardiometabolic diseases (1, 7-17)

A number of studies have compared the metabolic and vascular properties of different fat depots (18-27). Although multiple studies in rodents and humans have demonstrated that AT mitochondrial morphology, mass, and function are impaired in obesity (12, 28-40), studies that compared mitochondrial bioenergetics between different AT depots are scarce. We have recently shown that AT oxygen partial pressure is higher in abdominal (ABD) compared to femoral (FEM) subcutaneous AT in postmenopausal women, despite similar blood flow, suggesting that this might be due to lower oxygen consumption (41), which in turn may impact AT biology (1, 8, 9, 42). Thus, it remains to be established if mitochondrial respiration differs between AT depots in humans, whether this is due to cell-autonomous differences in mitochondrial respiration, and whether possible depot differences are apparent both in individuals with normal weight and obesity.

Therefore, the present study aimed to explore whether mitochondrial respiration differs between upper and lower body subcutaneous human multipotent adipose-derived stem cells (hMADS; primary outcome) and AT in postmenopausal women. We hypothesized that in vitro mitochondrial oxygen consumption rates and in vivo AT oxygen extraction are lower in upper body as compared with lower body AT. To test our hypothesis, we (i) performed functional experiments to determine mitochondrial respiration in differentiated ABD and FEM hMADS; (ii) assessed oxidative phosphorylation (OXPHOS) protein expression and mtDNA copy number in ABD and FEM AT as well as differentiated hMADS; and (iii) explored in vivo fractional oxygen extraction and carbon dioxide release across ABD and FEM subcutaneous AT.

## Methods

### Study Population

Twenty-three postmenopausal women (aged 50-65 years) with normal weight (NW, body mass index [BMI] 18-25 kg/m^2^; *n* = 13) or obesity (OB, BMI 30-40 kg/m^2^; *n* = 10) were recruited. All individuals underwent a medical evaluation during the screening visit. Exclusion criteria were smoking, cardiovascular disease, type 2 diabetes, liver or kidney malfunction, any chronic medical condition requiring the use of medication known to affect body weight, glucose and/or lipid metabolism, use of anti-inflammatory agents within 14 days prior to study start, intolerance/allergy to any of the test meal ingredients, blood donation 2 months prior to study completion, and marked alcohol consumption (> 14 alcoholic units/week). Premenopausal or perimenopausal women, defined as either regular periods or a period within the last 12 months prior to screening, were excluded.

The in vivo measurements were conducted at the National Institute for Health Research/Wellcome Trust Clinical Research Facility of the University of Birmingham/Queen Elizabeth Hospital Birmingham (Birmingham, UK). Participants were asked to arrive at the Clinical Research Facility after an overnight fast, having avoided strenuous exercise and alcohol consumption for at least 24 hours, on 3 occasions. Each of these study visits took place within 1 week of the previous visit, separated by at least 2 days.

The University of Birmingham Ethics committee and the UK Health Research Authority National Health System Research Ethics Committee approved the present study (approval no. 18/NW/0392). The study was performed according to the Declaration of Helsinki, and all participants provided written informed consent before taking part in the study procedures. The in vitro experiments and sample analyses were performed at Maastricht University Medical Center^+^ (Maastricht, the Netherlands).

### Screening

During the first study visit, body weight, height, waist (measured midway between the lower margin of the last palpable rib and the top of the iliac crest) and hip circumferences (measured at the level of the greater trochanters) were determined. Blood pressure and heart rate were measured using a standard oscillometric blood pressure monitor with an upper arm cuff. Next, we screened blood vessels in ABD and FEM AT using ultrasound to determine whether veins would be suitable for cannulation. Finally, a 5-point oral glucose tolerance test was performed. Briefly, an antecubital vein catheter was inserted, and a fasting blood sample (t = 0 minutes) was collected. Subjects were then asked to ingest 75 g of glucose in the form of a premade drink (113 mL of Polycal Liquid, Nutricia Ltd, Trowbridge, Wiltshire, UK), and blood samples were collected at t = 30, 60, 90 and 120 minutes.

### Adipose Tissue Biopsies

ABD and FEM AT needle biopsy specimens (∼0.5-1 g) were collected 6 to 8 cm lateral from the umbilicus and from the anterior site of the upper leg, respectively, under local anesthesia (1% lidocaine) after an overnight fast. Biopsy specimens were immediately rinsed with sterile saline and visible blood vessels were removed with sterile tweezers. The AT was fixed overnight in 4% paraformaldehyde and embedded in paraffin to determine adipocyte morphology. Another part was used for isolation of human multipotent adipose-derived stem (hMADS) cells, as described before (41). The remaining tissue was snap-frozen in liquid nitrogen and stored at −80 °C for gene/protein expression analysis.

### Adipocyte Morphology

Adipocyte morphology was assessed using the ABD and FEM AT biopsies, as described before (41). Histological sections (8 μm) were cut from paraffin-embedded tissue, mounted on microscope glass slides, and dried overnight in an incubator at 37 °C. Sections were stained with hematoxylin and eosin. Digital images were captured with the use of a Leica DFC320 digital camera (Leica, Rijswijk, Netherlands) at ×20 magnification (Leica DM3000 microscope; Leica). Computerized morphometric analysis (Leica QWin V3, Cambridge, England) of individual adipocytes was performed by measuring at least 200 adipocytes per sample.

### Oxygen Consumption in Differentiated hMADS

We obtained hMADS, an established human white adipocyte model (43), from ABD and FEM subcutaneous AT of the same participants who also underwent the in vivo measurements. Cells were seeded at a density of 2000 cells/cm^2^ and kept in proliferation medium for 7 days and thereafter in differentiation medium for 14 days. All experiments were performed on day 14 of the adipogenic differentiation. Paired ABD and FEM hMADS samples derived from 10 women with NW and 10 women with OB were used for these experiments.

The primary outcome of the present study, the oxygen consumption rate (OCR) that reflects mitochondrial respiration, was measured in differentiated hMADS cells using a XF96 analyzer (Seahorse Bioscience, North Billerica, MA) (41). Just before the measurement, cells were washed with unbuffered DMEM. A bioenergetics profile was determined using a 4-step analysis: (i) basal OCR was measured in medium containing (17.5 mM) glucose (Sigma) (calculated by the average respiration of 2 time points at the end of step 1); (ii) ATP turnover/production rate was determined by measuring OCR after inhibition of ATP synthase by oligomycin (1.4 μM; Sigma) (calculated by the average of respiration at the end of step 1 subtracted by the respiration after step 2); (iii) maximal mitochondrial respiratory capacity was assessed by measuring OCR after stimulation with the uncoupling agent carbonyl cyanide-*p*-trifluoromethoxyphenylhydrazone (0.3 μM; Sigma) (calculated by the respiration at the end of step 3 subtracted by the respiration after step 1); and (iv) nonmitochondrial respiration was assessed by measuring OCR after addition of complex I inhibitor: antimycin A/rotenone (1 μM; Sigma); proton leak was calculated by the average respiration at the end of the fourth step subtracting average at the end of the second step. Measurements were normalized for bicinchoninic acid (BCA) signal per well.

We also determined gene expression of several adipocyte differentiation markers: peroxisome proliferator-activated receptor γ (PPARγ), CCAAT-enhancer binding protein α (C/EBPα), fatty acid synthase (FAS), and Perilipin 1 (PLIN1). Therefore, total RNA was extracted from differentiated hMADS cells using TRIzol reagent (Invitrogen, Breda, Netherlands), and SYBR-Green–based real-time PCRs were performed using an iCycler (Bio-Rad, Veenendaal, Netherlands). Results were normalized to 18S ribosomal RNA.

### OXPHOS Protein Expression in Adipose Tissue and Differentiated hMADS

OXPHOS protein content was determined in paired ABD and FEM AT biopsies from 10 postmenopausal women with NW and 8 with OB, as well as paired differentiated ABD and FEM hMADS using Western blot analyses. A cocktail of mouse monoclonal antibodies directed against human OXPHOS complexes was used (Abcam Cat# ab110411, RRID:AB_2756818). The specific proteins were detected using secondary antibodies conjugated with IRDye800 and were quantified using the CLx Odyssey Near Infrared Imager (Li-COR, Westburg).

### Mitochondrial DNA Copy Number in Adipose Tissue and Differentiated hMADS

Relative amounts of nuclear DNA and mitochondrial (mt)DNA were determined by quantitative PCR, as previously described (44, 45). The ratio of mtDNA to nuclear DNA reflects the tissue concentration of mtDNA per cell. Briefly, an mtDNA fragment within the *ND1* gene (also known as *MT-ND1*) for NADH dehydrogenase subunit 1 was used for quantification of mtDNA (45, 46). A region of the nuclear gene for lipoprotein lipase (*LPL*), was used to normalize results (44). The ratio of *ND1* to *LPL* copy number reflects the tissue concentration of mitochondria per cell (44).

### In Vivo Measurements

#### Arterio-venous concentration differences across adipose tissue

Arterio-venous concentration differences of oxygen and carbon dioxide, and several metabolites across ABD and FEM subcutaneous AT were assessed, as described previously (47, 48). Briefly, selective venous catheterization of one the branches of the superficial epigastric veins (*vena epigastrica superficialis*; draining ABD AT) was performed (48-50). Next, a superficial branch of the great saphenous vein (*vena saphena magna*; draining FEM subcutaneous AT) was cannulated (51). Finally, a catheter was inserted into the radial artery. Sixty minutes after the cannulation procedures (allowing participants to relax), blood samples were taken simultaneously from the 3 sites (arterial, ABD, and FEM) at 2 different time points, separated by 30 minutes, under fasting conditions. Thereafter, study participants were provided a high-fat mixed-meal (2.6MJ, consisting of 61.2 E% fat, 32.6 E% carbohydrates, and 6.3 E% protein) to be consumed within 5 to 10 minutes, and arterial blood samples were collected at t = 30, 60, 90, 120, 180, and 240 minutes. After study completion, all catheters were removed, and the participants were given a meal. Due to the technical difficulties associated with cannulation of and sampling from the small veins in ABD and FEM AT, fasting adipose venous samples were only obtained in a subgroup of the study population (n = 15; 9 NW and 6 OB). Therefore, in vivo AT depot differences in oxygen fractional extraction and fractional carbon dioxide release were explored in this subgroup of individuals.

#### Adipose tissue blood flow

Fasting AT blood flow (ATBF) was measured in ABD and FEM AT, approximately 5 minutes after blood sampling, using a Doppler ultrasound technique, as previously described (21). ATBF was measured using a Philips CX50 ultrasound system (Philips Ultrasound, Bothell, USA), and ATBF was calculated as described previously (21).

#### Body composition

A dual-energy x-ray absorptiometry (DXA) scan was performed after an overnight fast to determine body composition and body fat percentage (Lunar iDXA, GE Healthcare) (52).

### Biochemical Analyses

Blood samples were drawn into heparinized syringes, centrifuged at 4 °C at 1000*g*, and plasma was immediately frozen and stored at −80 °C until analysis. Plasma glucose (ABX Pentra Glucose HK CP, ABX Diagnostics, Montpellier, France), NEFA (WAKO NEFA-HR ACS-ACOD method, WAKO Chemicals GmbH, Neuss, Germany) and lactate were determined on a Cobas Pentra C400 (Horiba Europe GmbH, Son, the Netherlands). Plasma insulin was assessed using a Human Insulin ELISA kit (Meso Scale Discovery, Gaithersburg, USA). Blood gases were analyzed using the point-of-care blood gas analyzer (Roche Cobas b221, Roche Diagnostics, West Sussex, United Kingdom) at the bedside.

### Calculations

Insulin resistance was calculated using the updated computer model-based homeostatic model assessment (HOMA) method (53). The net uptake or release or extraction of blood gases and metabolites across ABD and FEM AT were calculated using the arterio-venous differences. Fractional extraction (FE) was calculated by dividing the arterio-venous concentration difference by the arterial concentration [FE = ([arterial − venous concentration]/arterial concentration) × 100%]. Fractional release (FR) was calculated by dividing the veno-arterial concentration difference by the arterial concentration [FR = ([venous − arterial concentration]/arterial concentration) × 100%] (50, 54). For metabolite data obtained from the meal test, the (incremental) area under the curve (i)AUC was calculated using the trapezoid rule.

### Statistical Analysis

AT depot differences were tested using Student paired *t* tests (Wilcoxon signed rank tests in case of skewed data, as determined by the Shapiro-Wilk test), while differences between NW and OB individuals were tested using Student unpaired *t* tests (Mann-Whitney test in case data were not normally distributed). Given the exploratory nature of the in vivo measurements of oxygen fractional extraction and carbon dioxide fractional release, performed in a relatively low number of individuals with NW and OB in whom paired measurements were available, we did not perform statistical analyses but rather present descriptive data. GraphPad Prism version 8 for Windows was used to perform statistics, and *P* < .05 was considered as statistically significant and *P* < .10 as a tendency for significance. Data are presented as mean ± standard error of the mean.

## Results

### Subject Characteristics

Participants’ characteristics are shown in Table 1. As expected, BMI, body fat percentage, waist and hip circumferences, visceral fat mass, abdominal and femoral subcutaneous fat mass were higher in OB than NW individuals (all *P* < .001), whereas fasting insulin concentration (*P* = .053) and HOMA2-IR (*P* = .050) tended to be higher in the OB vs NW group.

**Table 1.** Characteristics of the study participants

	Normal weight (n = 13)	Obesity (n = 10)	*P* value
Age, years	56.6 ± 1.5	56.6 ± 1.1	.994
BMI, kg/m^2^	22.9 ± 0.4	34.5 ± 0.9	<.001
Waist circumference, cm	78.6 ± 2.2	105.1 ± 4.2	<.001
Hip circumference, cm	95.0 ± 2.1	125.2 ± 7.3	<.001
Waist-to-hip ratio	0.83 ± 0.02	0.86 ± 0.05	.538
Visceral fat mass, g	350 ± 88	1272 ± 140	<.001
Abdominal fat mass, kg	9.91 ± 0.98	23.22 ± 1.91	<.001
Leg fat mass, kg	7.57 ± 0.60	15.29 ± 1.18	<.001
Fasting glucose, mmol/L	4.97 ± 0.10	5.16 ± 0.20	.404
2-Hour glucose, mmol/L	5.09 ± 0.20	4.99 ± 0.30	.775
Fasting insulin, pmol/L	25.40 ± 4.00	48.60 ± 12.90	.053
HOMA2-IR	0.47 ± 0.1	0.92 ± 0.3	.050
SBP, mmHg	120.9 ± 3.9	131.3 ± 4.3	.098
DBP, mmHg	76.2 ± 3.1	80.6 ± 3.1	.356
Abdominal adipocyte diameter, μm	64.4 ± 1.4	72.1 ± 1.5	.014
Femoral adipocyte diameter, μm	63.6 ± 1.2	73.7 ± 1.6	.001
Fasting abdominal ATBF, mL/min	8.4 ± 2.1	11.2 ± 2.9	.459
Fasting femoral ATBF, mL/min	5.3 ± 1.5	6.4 ± 2.1	.755

Data are mean ± SEM. Abbreviations: ATBF, adipose tissue blood flow; BMI, body mass index; DBP, diastolic blood pressure; HOMA2-IR, homeostasis model assessment 2 for insulin resistance; SBP, systolic blood pressure.

Furthermore, both abdominal (*P* = .014) and femoral (*P* = .001) subcutaneous adipocyte size was higher in individuals with OB than NW, with no depot differences in adipocyte size (Table 1).

### Oxygen Consumption Rates in Differentiated hMADS Derived From Abdominal and Femoral Subcutaneous Adipose Tissue

**Figure 1.**
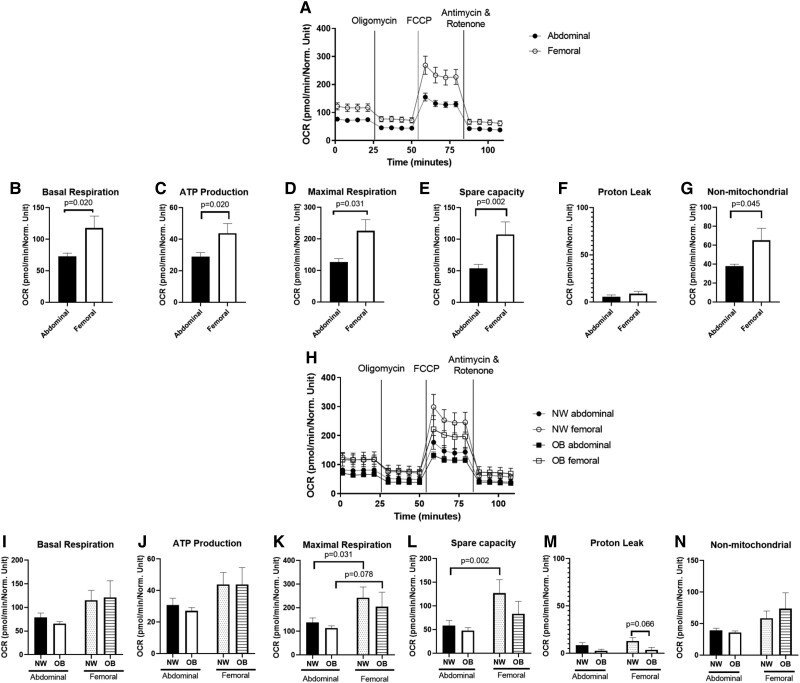
Oxygen consumption rates (OCR) in abdominal and femoral subcutaneous differentiated human multipotent adipose-derived stem cells (hMADS). OCR was determined in hMADS derived from the total group of women with normal weight and obesity (panels A-G; n = 18-20) as well as for the normal weight and obese groups separately (Panels H-N; n = 8-10 per group). Data are expressed as mean ± SEM. Abbreviations: NW, normal weight; OCR, oxygen consumption rate; OB, obesity.

Since AT consists of multiple cells, we determined OCR in differentiated hMADS. OCR was significantly lower in ABD compared with FEM hMADS (Fig. 1A-1G). More specifically, basal respiration (*P* = .020, Fig. 1B), ATP production (*P* = .020, Fig. 1C), maximal respiration (*P* = .031, Fig. 1D), and spare capacity (*P* = .002, Fig. 1E) were lower in ABD compared to FEM hMADS. Proton leak did not differ between ABD and FEM adipocytes (*P* = .306, Fig. 1F). Nonmitochondrial OCR was higher in FEM than ABD hMADS (*P* = .045, Fig. 1G)

OCRs in hMADS from women with NW and OB showed a similar pattern as those for the total group (Fig. 1H-1N). Specifically, we found lower maximal respiration in ABD compared to FEM hMADS derived from NW (*P* = .031) and OB (*P* = .078) individuals (Fig. 1K). Moreover, spare capacity was lower in ABD than FEM hMADS derived from individuals with NW (*P* = .002, Fig. 1L). Gene expression of the adipocyte differentiation markers peroxisome proliferator-activated receptor γ (PPARγ), CCAAT-enhancer binding protein α (C/EBPα), Perilipin 1 (PLIN1) and fatty acid synthase (FAS) was not different between ABD and FEM adipocytes (data not shown), indicating that the differences in OCR are not due to differences in adipocyte differentiation.

### OXPHOS Protein Expression in Abdominal and Femoral Subcutaneous Adipose Tissue and Differentiated hMADS

#### Adipose tissue

**Figure 2.**
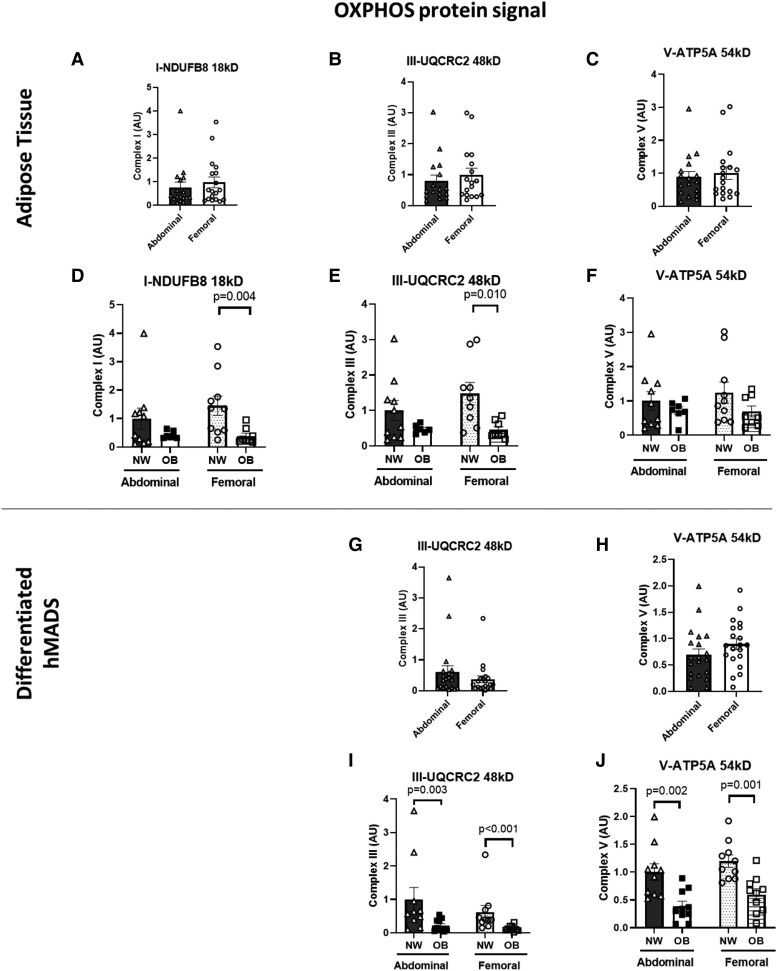
OXPHOS protein expression in abdominal and femoral subcutaneous adipose tissue and differentiated human multipotent adipose-derived stem cells (hMADS). Adipose tissue OXPHOS expression is shown for the total group of women with normal weight and obesity (panels A-C; n = 18) as well as for the normal weight and obese groups separately (panels D-F). Differentiated hMADS OXPHOS expression is shown for the total group of women with normal weight and obesity (panels G and H; n = 20) as well as for the normal weight and obese groups separately (panels I and J; abdominal NW, n = 10; abdominal OB, n = 10; femoral NW, n = 10; femoral OB, n = 10). Data are expressed as mean ± SEM. Abbreviations: AU, arbitrary units; NW, normal weight; OB, obesity.

To further examine depot differences in the oxidative machinery, we measured OXPHOS protein complexes in paired ABD and FEM AT biopsies. OXPHOS complex I, complex III, and complex V were not different in ABD compared to FEM AT (Fig. 2A-2C and Supplementary Fig. S1). However, protein expression of OXPHOS complexes I, III, and V were consistently lower in FEM AT in those with OB compared to NW, with significantly lower protein expression of OXPHOS complex I (*P* = .004) and III (*P* = .010) in femoral AT in individuals with OB (Fig. 2D-2F and Supplementary Fig. S1). Although protein expression of OXPHOS complexes seemed lower in ABD AT in OB compared to NW individuals, this did not reach statistical significance. Adipocyte size was comparable between ABD and FEM AT, but adipocytes were significantly larger in both AT depots in individuals with OB than NW (Table 1).

#### Differentiated hMADS

No differences in OXPHOS protein expression were found between ABD and FEM hMADS (Fig. 2G and 2H). However, protein expression of OXPHOS complex III was lower in FEM (*P* < .001) as well as ABD (*P* = .003) hMADS derived from OB compared to NW individuals (Fig. 2I). Similarly, OXPHOS complex V expression was lower in FEM (*P* = .001) and ABD (*P* = .002) hMADS derived from individuals with OB compared to those with NW (Fig. 2J). We were not able to detect protein expression of CI and CII in differentiated hMADS.

### mtDNA Copy Number in Abdominal and Femoral Subcutaneous Adipose Tissue and Differentiated hMADS

#### Adipose tissue

**Figure 3.**
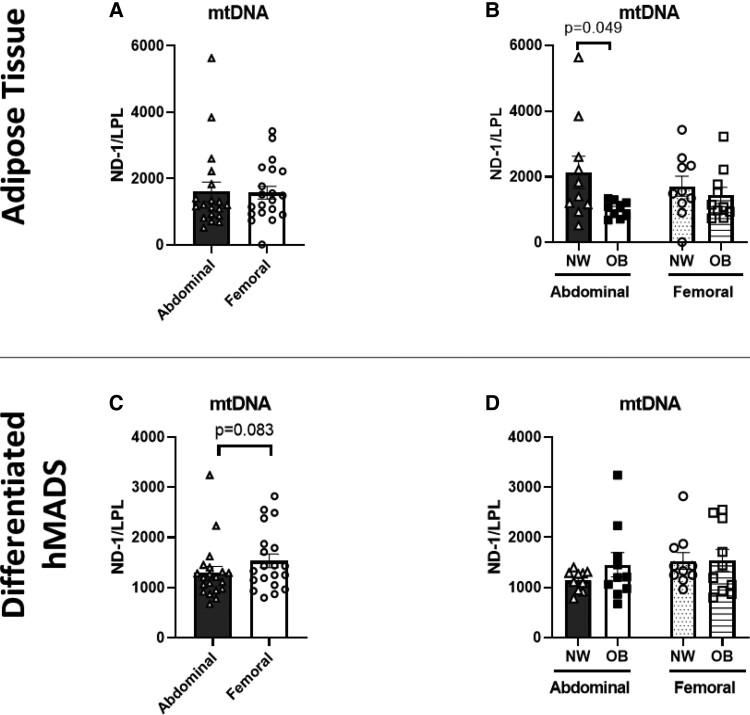
Mitochondrial (mt)DNA copy number in abdominal and femoral adipose tissue and differentiated human multipotent adipose-derived stem cells (hMADS). The mtDNA copy number was determined in adipose tissue in the total group of women with normal weight and obesity (panel A, n = 20) as well as for the normal weight and obese groups separately (panel B). The hMADS mtDNA copy number was assessed for the total group of women with normal weight and obesity (panel C, n = 20) as well as for the hMADS derived from the normal weight and obese groups separately (panel D; abdominal NW, n = 10; abdominal OB, n = 9; femoral NW, n = 10; femoral OB, n = 10). Data are expressed as mean ± SEM. Abbreviations: NW, normal weight; OB, obesity.

No differences in mtDNA content were found between ABD and FEM AT (*P* = .891, Fig. 3A). Furthermore, mtDNA copy number was lower in ABD AT from individuals with OB compared to those with NW (*P* = .049), while no differences were found for FEM AT (*P* = .488, Fig. 2B).

#### Differentiated hMADS

The mtDNA copy number tended to be lower in ABD compared to FEM hMADS in the total group (*P* = .083, Fig. 3C). For the NW and OB groups separately, no significant differences in mtDNA copy number were found between ABD and FEM hMADS (Fig. 3D).

### Plasma Metabolite Concentrations

**Figure 4.**
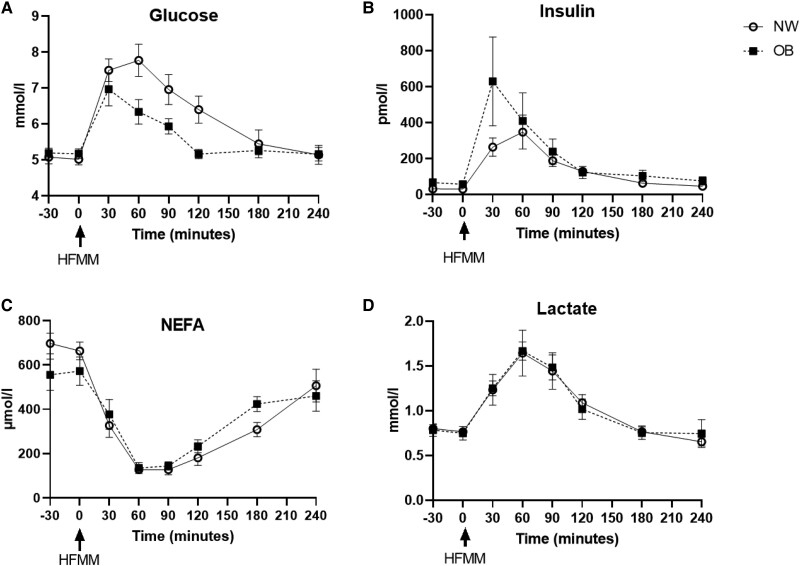
Fasting and postprandial plasma metabolites concentrations in arterial blood from postmenopausal women with normal weight (*n* = 9) and obesity (*n* = 6). A high-fat mixed-meal (HFMM) was ingested at t = 0 minutes. A, glucose; B, insulin; C, nonesterified fatty acids; D, lactate. Data are expressed as mean ± SEM. Abbreviations: NW, Normal weight; OB, obesity.

To phenotype the study participants in more detail, we determined arterial plasma concentrations of metabolites after an overnight fast and following the ingestion of a high-fat mixed-meal (HFMM) over a period of 240 minutes. No significant differences between NW and OB groups were found for circulating fasting glucose (*P* = .586), nonesterified fatty acids (NEFA) (*P* = .142), and lactate (*P* = .232) (Fig. 4). Fasting insulin tended to be higher in OB vs NW (*P* = .057). No significant differences in postprandial insulin, NEFA, and lactate were found between groups, whereas the postprandial increase in glucose concentrations was higher in participants with NW compared to those with OB (iAUC/min, *P* = .018) (Fig. 4).

### In Vivo Oxygen Extraction and Carbon Dioxide Release Across Abdominal and Femoral Subcutaneous Adipose Tissue

**Figure 5.**
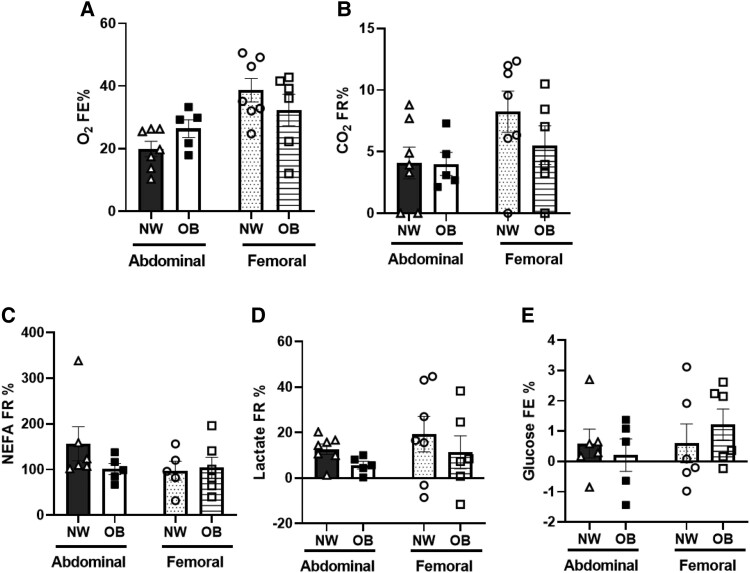
Fractional release or extraction of blood gases and metabolites across subcutaneous abdominal and femoral adipose tissue in postmenopausal women with normal weight (*n* = 9) and obesity (*n* = 6) under fasting conditions. A, Fractional extraction (FE) of oxygen; B, fractional release (FR) of carbon dioxide; C, fractional release of nonesterified fatty acids; D, fractional release of lactate; and E, fractional extraction of glucose. Data are expressed as mean ± SEM. Abbreviations: CO_2_, carbon dioxide; NEFA, nonesterified fatty acids; NW, normal weight; O_2_, oxygen; OB, obesity.

The fractional extraction (FE) of oxygen was lower across ABD as compared to FEM AT both in participants with NW (ABD: 19.9 ± 2.4 vs FEM: 38.7 ± 3.8 and OB (ABD: 26.4 ± 2.8 vs FEM: 32.4 ± 5.1) (Fig. 5A). This was accompanied by a lower fractional release (FR) of carbon dioxide across ABD than FEM AT both in people with NW (ABD: 4.0 ± 1.3 vs FEM: 8.2 ± 1.7) and OB (ABD: 4.09 ± .9 vs FEM: 5.5 ± 1.6) (Fig. 5B). The fractional release of NEFA and lactate (Figs. 5C and 5D), and fractional extraction of glucose (Fig. 5E) did not show clear depot differences. No significant differences in adipose tissue blood flow were found between ABD and FEM AT (Table 1).

## Discussion

In the present study, we examined the oxidative signatures of ABD and FEM subcutaneous differentiated hMADS as well as AT in postmenopausal women by investigating the oxygen consumption rates in differentiated ABD and FEM hMADS derived from individuals with normal weight or obesity, OXPHOS protein expression and mtDNA copy number in hMADS and AT, as well as by exploring in vivo metabolic fluxes across these AT depots. Our findings demonstrate lower in vitro mitochondrial oxygen consumption rates in abdominal compared to femoral differentiated hMADS, and lower in vivo fractional oxygen extraction and carbon dioxide release across abdominal than femoral AT, with no differences in adipocyte morphology, OXPHOS protein expression, and mtDNA content.

We found that oxygen consumption rates were lower in differentiated hMADS derived from ABD compared to FEM AT. Specifically, we found significantly lower basal respiration, ATP production, maximal respiration, and spare capacity in ABD vs FEM differentiated hMADS. Since previous studies have shown that aspects of mitochondrial function are impaired in obesity, we further compared depot differences in OCRs in women with normal weight or obesity separately. In line with the data for the total group, subgroup analysis showed that all functional readouts related to mitochondrial respiration tended to be lower in differentiated ABD compared to FEM hMADS. Statistical significance was, however, only reached for maximal respiration, which is likely due to limited statistical power for the secondary analyses in these subgroups. Adipocyte differentiation markers were not significantly different between ABD and FEM hMADS, suggesting that these findings are not due to differences in adipocyte differentiation.

Human in vivo data on AT oxygen consumption remain sparse (9, 55), which is at least partially due to the technical challenges related to the execution of these measurements in humans. We investigated whether depot differences in the oxygen consumption rates of human hMADS may translate into lower in vivo oxygen fractional extraction across ABD AT. Indeed, we found that the in vivo fractional extraction of oxygen was lower across ABD compared to FEM subcutaneous AT. In line, carbon dioxide fractional release was lower across ABD vs FEM AT. Furthermore, we found no significant depot differences in adipocyte size and ATBF. Strikingly, these findings suggest that depot differences in oxygen extraction in vivo are maintained in vitro after differentiation of hMADS derived from these different fat depots. Taken together, the present study demonstrates that both human ABD hMADS oxygen consumption and in vivo oxygen fractional extraction across ABD AT are lower than in FEM hMADS and AT, respectively, in postmenopausal women. These findings may explain the higher oxygen partial pressure (pO_2_) in ABD than FEM AT that we recently found in postmenopausal women (41), which in turn might contribute to differences between upper and lower body AT function in humans (9, 55). Additionally, we found no differences in the in vivo fractional extraction or release of glucose, NEFA, and lactate between ABD and FEM AT. Previous studies have also shown similar (24) or higher NEFA release across ABD compared to FEM subcutaneous AT in healthy, younger individuals (20, 22).

To examine the AT oxidative signatures in more detail, we also determined OXPHOS protein concentrations and mtDNA copy numbers, both in ABD and FEM differentiated hMADS and AT. The OXPHOS system is crucial in aerobic energy production by transferring high-energy electrons (derived from substrate oxidation) through the electron transport chain in the inner mitochondrial membrane (complexes I-IV of the OXPHOS system), thereby generating an electrochemical gradient across the inner membrane which is then utilized by complex V (ATP synthase) to phosphorylate ADP to ATP (10, 56). Interestingly, we found no significant differences in the expression of the different OXPHOS complexes and mtDNA content between ABD and FEM differentiated hMADS and AT, although mtDNA content tended to be lower in ABD hMADS. These data suggest that expression of OXPHOS and mtDNA copy number are not directly associated with functional measurements of oxygen consumption in human hMADS and AT. Indeed, a comparable dissociation between OCR and mitochondrial content has been shown in mature ABD subcutaneous adipocytes isolated from AT biopsies obtained from individuals across a broad BMI range (from normal weight to morbid obesity) (40). A possible explanation for these depot differences in mitochondrial respiration may be related to inflammation. It is well established that mitochondria are important regulators of inflammatory responses (57). On the other hand, inflammatory factors may impair mitochondrial biogenesis and function (58). Interestingly, we have recently demonstrated that differentiated hMADS derived from ABD AT indeed showed increased gene expression and secretion of several pro-inflammatory factors compared to hMADS derived from FEM AT (59).

In addition to AT depot differences in the total study population, we also investigated depot differences in the AT oxidative machinery between individuals with NW and OB. Here, we demonstrate that the protein expression of OXPHOS complexes was significantly lower in FEM AT (complexes I, III) and ABD and FEM differentiated hMADS (complexes III, V) derived from postmenopausal women with OB compared to those with NW. Complex V protein levels were significantly lower in hMADS derived from both fat depots in women with OB but did not reach statistical significance for AT. The latter may be explained by the limited statistical power for these secondary subgroup analyses, but it may also relate to the fact that AT consists of multiple cell types in addition to adipocytes, and that factors in the adipose tissue microenvironment differ from those in cell culture conditions (ie, oxygen availability, temperature, fatty acid composition). Our findings extend previous observations showing lower OXPHOS expression (36, 38, 39) and a lower in vivo ABD subcutaneous AT oxygen consumption in obesity (33). In agreement with most (36, 39, 45) but not all (36, 40) findings, we also found that ABD AT mtDNA content was significantly lower in individuals with OB than NW. In contrast, no group differences in mtDNA content were found for FEM AT. Interestingly, we did not observe any differences in OCR between differentiated hMADS derived from individuals with NW and OB. The latter finding seems in contrast with previous studies showing impaired OCR in adipocytes from individuals with OB compared to NW, independent of fat cell size (36, 40). Importantly, however, it is important to emphasize that previous studies measured OCR in mature adipocytes isolated from AT biopsies. Therefore, it cannot be excluded that these conflicting findings are due to differences in experimental conditions or study populations compared to the present study. Based on fasting insulin concentrations and HOMA2-IR, the individuals with OB tended to be more insulin resistant, although no significant differences were found in circulating metabolites between the NW and OB groups under fasting conditions. Furthermore, the postprandial decrease in arterial NEFA concentrations was more pronounced in the NW group, indicative of higher AT insulin sensitivity. Further studies are warranted to examine AT depot differences in the mitochondrial respiration in different populations, varying in age, sex, metabolic status, and obesity duration.

A strength of the present study is that we phenotyped the study participants in detail and combined functional measurements in ABD and FEM differentiated hMADS, in vivo measurements across different AT depots, and expression analyses in ABD and FEM AT and differentiated hMADS in the same individuals. However, this study also has several limitations. First, we performed analyses in differentiated hMADS. Although the lower OCR in differentiated hMADS from abdominal compared to femoral adipose tissue suggest that cell-autonomous differences contribute to AT depot differences in mitochondrial respiration, ex vivo mitochondrial respiration measurements in fresh tissue (whole AT, isolated mature adipocytes and/or isolated mitochondria) would have provided further insight into the contribution of cell-autonomous processes that are driven by intrinsic mechanisms within individual cells and external influences (ie, microenvironment) to the observed depot differences in mitochondrial respiration. Future studies in fresh AT and/or isolated mature adipocytes from ABD and FEM AT, including assessment of enzymatic activity of OXPHOS, are therefore warranted to investigate this in more detail. Furthermore, it would be interesting to investigate mitochondrial dynamics (eg, fission and fusion) (60, 61), as well as the underlying molecular mechanism(s) for depot differences in mitochondrial respiration in more detail in future studies. In addition, the sample size for the in vivo measurements across ABD and FEM subcutaneous AT is relatively small due to the technical difficulties to cannulate the small veins in these fat depots, as well as recruitment issues due to the COVID-19 pandemic. Moreover, although we were able to calculate fractional extraction and release across fat depots, in vivo fluxes across AT could not be quantified due to the lack of quantitative measures of blood flow per unit AT due to the production stop of ^133^Xe. Therefore, the present in vivo findings on AT depot differences in oxygen fractional extraction and carbon dioxide fractional release are exploratory in nature and require confirmation in a larger study population.

*In summary,* our findings demonstrate that upper and lower body AT are characterized by distinct oxidative signatures in postmenopausal women with normal weight and obesity, which seem independent of adipocyte size. Mitochondrial respiration rates are lower in differentiated hMADS from ABD than FEM AT in postmenopausal women, with no significant depot differences in OXPHOS protein expression and mtDNA content. Explorative data showing lower in vivo oxygen fractional extraction and higher carbon dioxide fractional release across abdominal compared to femoral subcutaneous AT in the same individuals support these findings. In addition, we found lower OXPHOS protein expression in AT and hMADS in women with obesity vs normal weight. Future studies are warranted to investigate functional differences between upper and lower body AT in different populations, and further investigate the relationship between mitochondrial function, morphology and dynamics, and the metabolic and inflammatory phenotype in AT as well as at the whole-body level.

## Data Availability

The data generated and analyzed in the present study are included in this published article and are available from the corresponding author upon reasonable request.

## Prior Presentation

A part of this study was presented at the European Association for the Study of Diabetes (EASD) Annual Meeting in 2022.

## Abbreviations

ABD: abdominal
AT: adipose tissue
ATBF: adipose tissue blood flow
BMI: body mass index
FE: fractional extraction
FEM: femoral
FR: fractional release
hMADS: human multipotent adipose-derived stem cells
HOMA-IR: homeostatic model assessment for insulin resistance
iAUC: incremental area under the curve
mtDNA: mitochondrial DNA
NEFA: nonesterified fatty acids
NW: normal weight
OB: obesity
OCR: oxygen consumption rate
OXPHOS: oxidative phosphorylation
